# Supplementary figures and images for: The genetic and dietary landscape of the muscle insulin signalling network
Source: eLife. 2024 Feb 8;12:RP89212. doi: 10.7554/eLife.89212 (PMC10942587; doi:10.7554/eLife.89212)

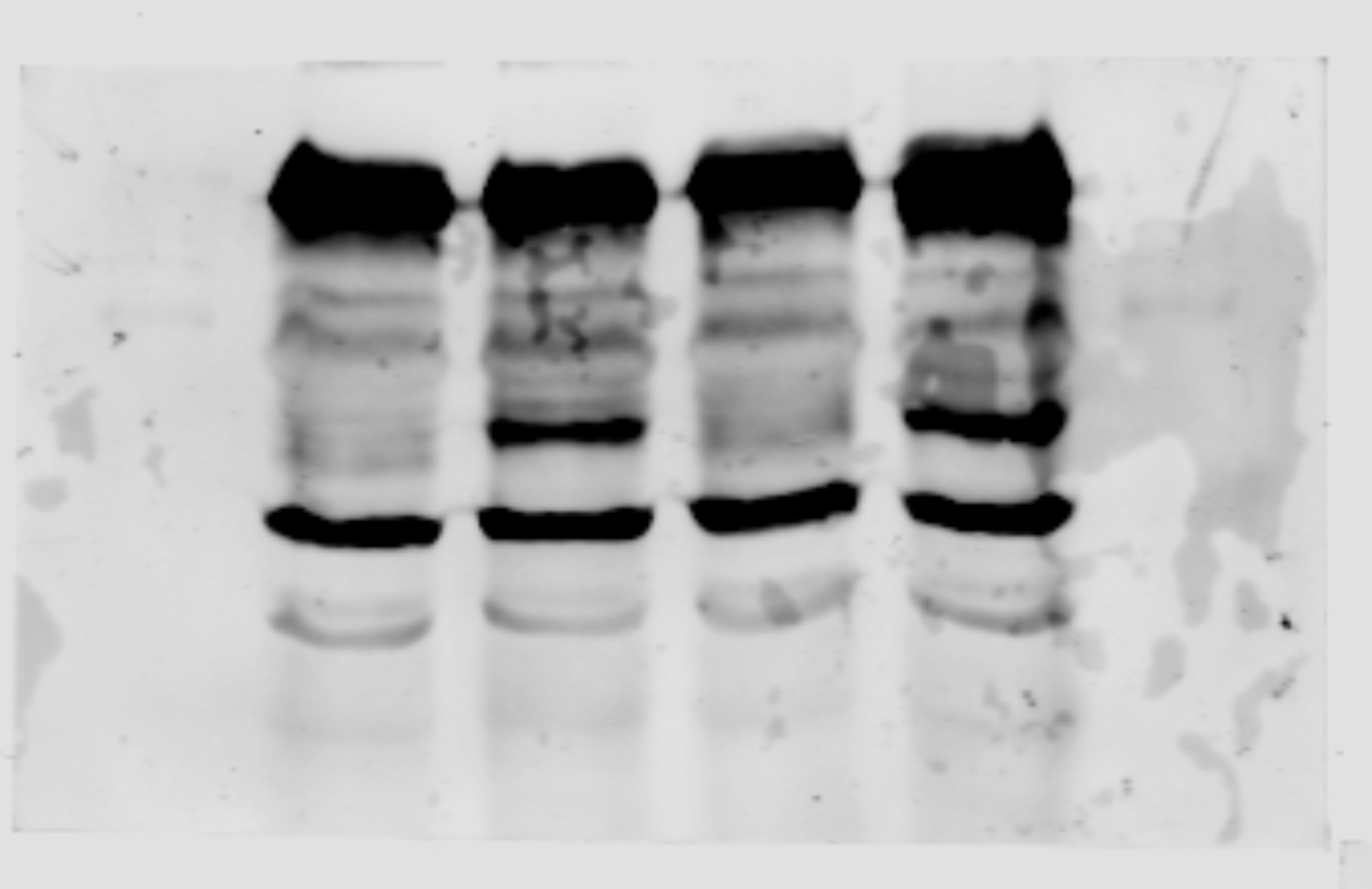

Supplement: Figure 6—figure supplement 2—source data 1. [file elife-89212-fig6-figsupp2-data1.zip › F01_Pfkfb3.tif]

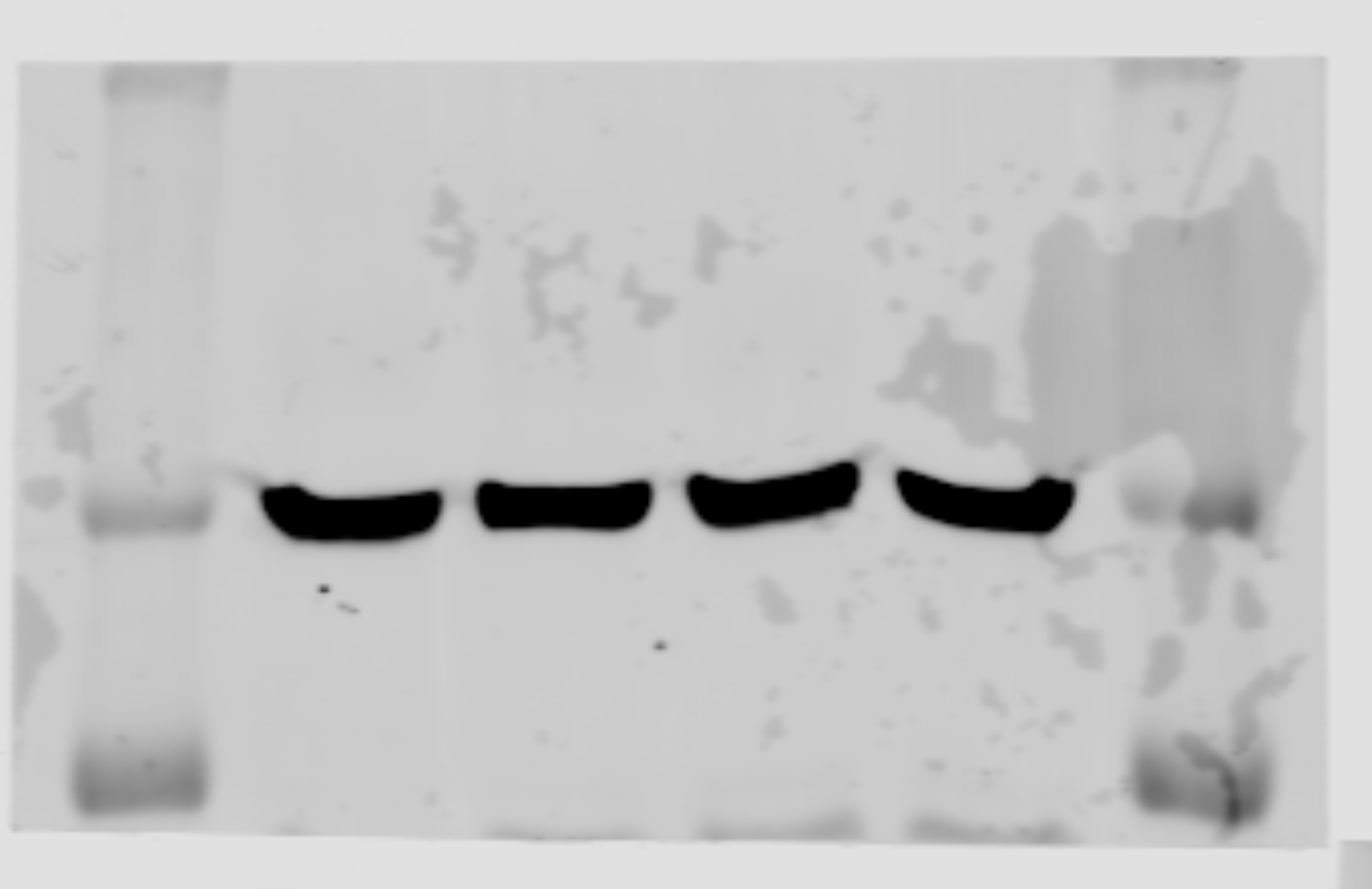

Supplement: Figure 6—figure supplement 2—source data 1. [file elife-89212-fig6-figsupp2-data1.zip › F02_tubulin.tif]

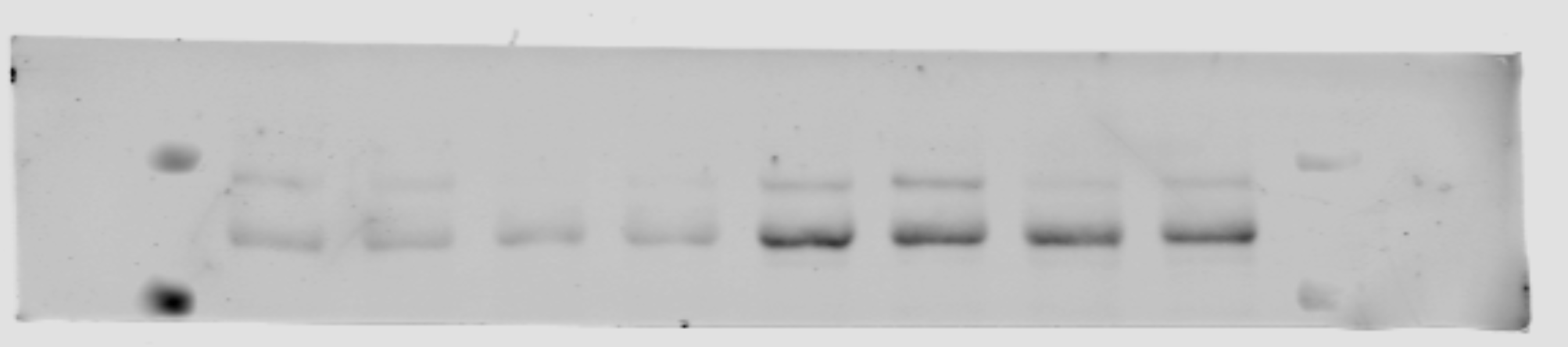

Supplement: Figure 6—figure supplement 2—source data 1. [file elife-89212-fig6-figsupp2-data1.zip › F1_pGsk3.tif]

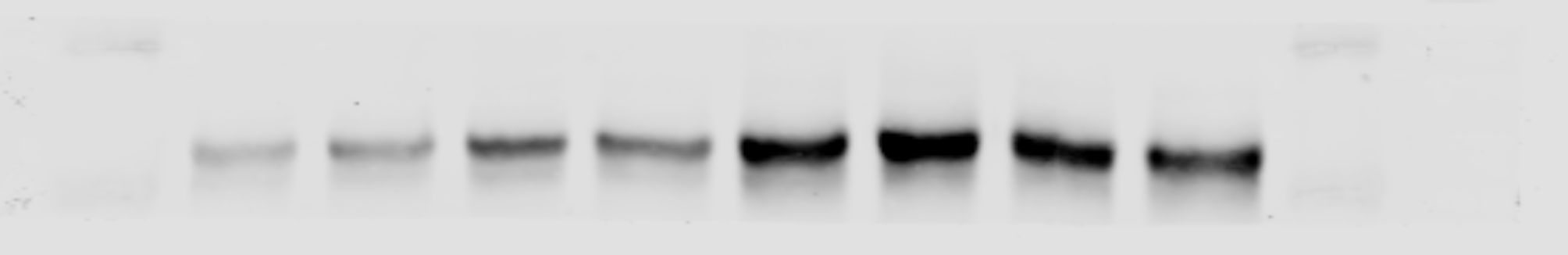

Supplement: Figure 6—figure supplement 2—source data 1. [file elife-89212-fig6-figsupp2-data1.zip › F2_pPRAS40.tif]

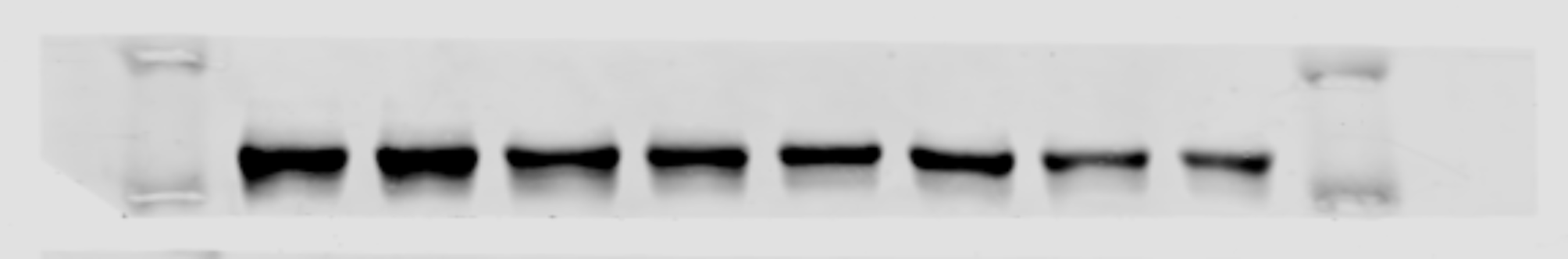

Supplement: Figure 6—figure supplement 2—source data 1. [file elife-89212-fig6-figsupp2-data1.zip › F3_tPRAS40.tif]

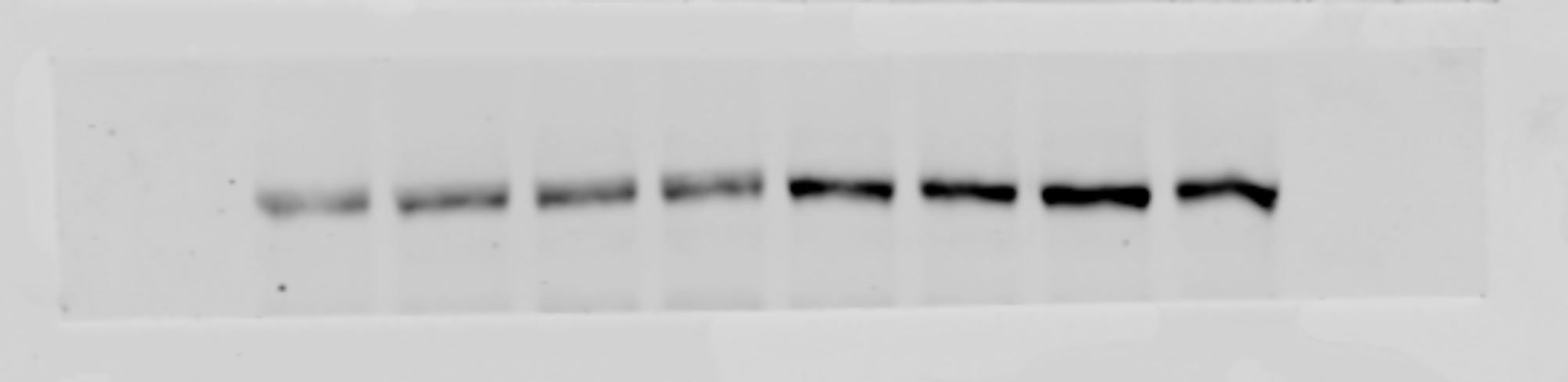

Supplement: Figure 6—figure supplement 2—source data 1. [file elife-89212-fig6-figsupp2-data1.zip › F4_pS473Akt.tif]

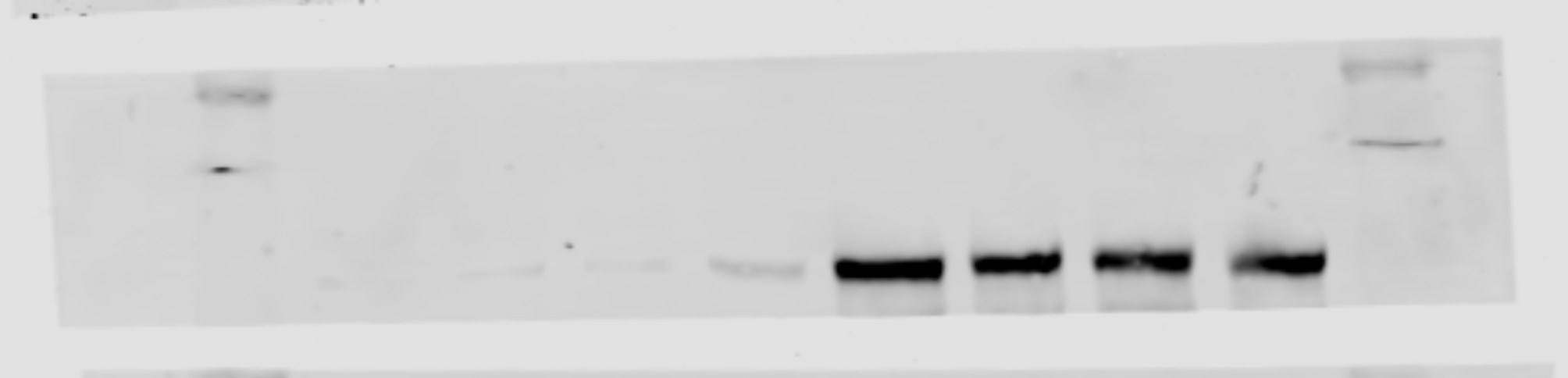

Supplement: Figure 6—figure supplement 2—source data 1. [file elife-89212-fig6-figsupp2-data1.zip › F5_pT308Akt.tif]

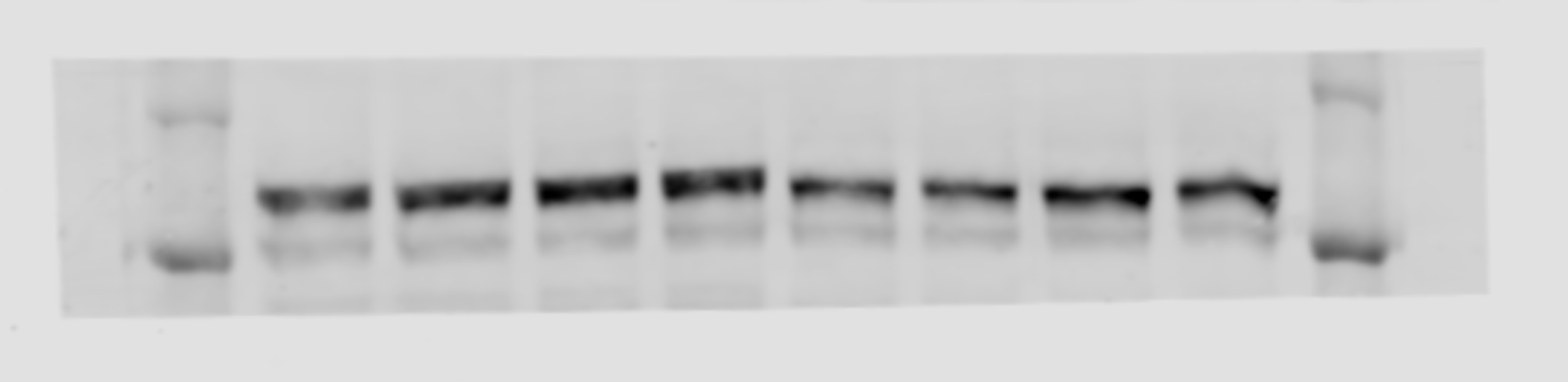

Supplement: Figure 6—figure supplement 2—source data 1. [file elife-89212-fig6-figsupp2-data1.zip › F6_tAkt.tif]

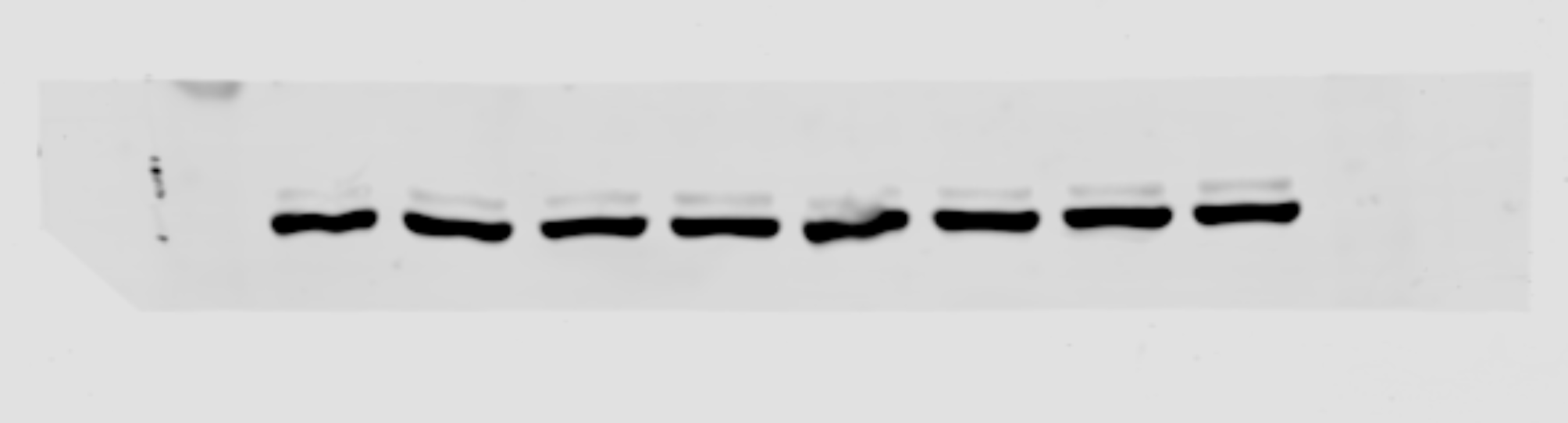

Supplement: Figure 6—figure supplement 2—source data 1. [file elife-89212-fig6-figsupp2-data1.zip › F7_1433.tif]

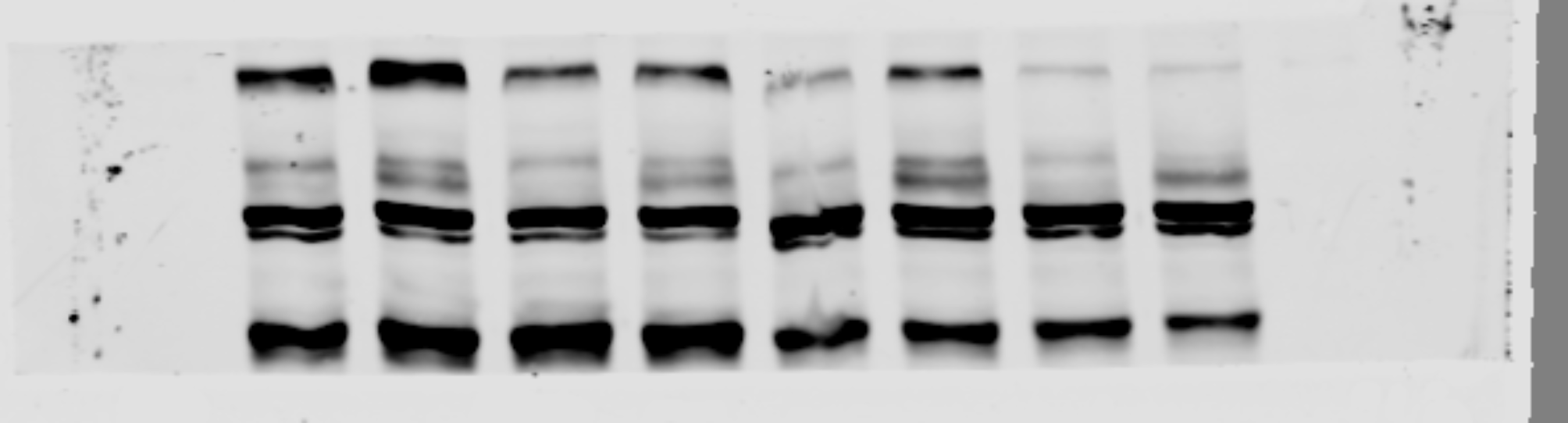

Supplement: Figure 6—figure supplement 2—source data 1. [file elife-89212-fig6-figsupp2-data1.zip › F8_PFKFB3_aTubulin.tif]
