# Supplementary material for: The genetic and dietary landscape of the muscle insulin signalling network
Source: eLife. 2024 Feb 8;12:RP89212. doi: 10.7554/eLife.89212 (PMC10942587; doi:10.7554/eLife.89212)
Supplement: Figure 6—figure supplement 2—source data 2. [file elife-89212-fig6-figsupp2-data2.zip › Fig6S2_source_data_2.pdf]

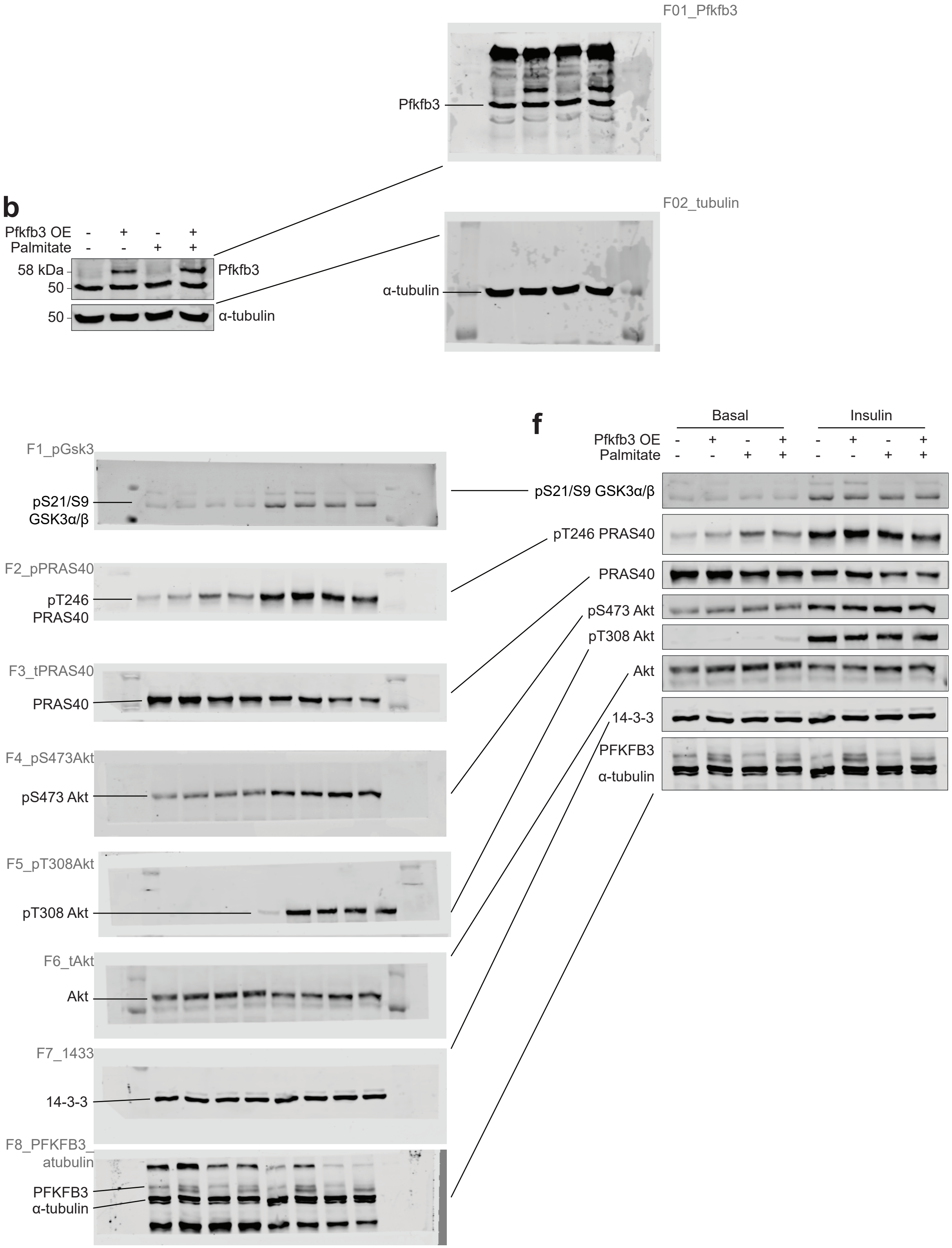

F01_Pfkfb3
Pfkfb3
b
Pfkfb3 OE - + - +
Palmitate - - + +
58 kDa
50
Pfkfb3
50
α-tubulin
F02_tubulin
α-tubulin
f
Basal
Insulin
Pfkfb3 OE - + - + - + - +
Palmitate - - + + - - + +
pS21/S9 GSK3α/β
pT246 PRAS40
PRAS40
pS473 Akt
pT308 Akt
Akt
14-3-3
PFKFB3
α-tubulin
F1_pGsk3
pS21/S9
GSK3α/β
F2_pPRAS40
pT246
PRAS40
F3_tPRAS40
PRAS40
F4_pS473Akt
pS473 Akt
F5_pT308Akt
pT308 Akt
F6_tAkt
Akt
F7_1433
14-3-3
F8_PFKFB3_
atubulin
PFKFB3
α-tubulin
